# Supplementary material for: The Systems Biology Research Tool: evolvable open-source software
Source: BMC Syst Biol. 2008 Jun 29;2:55. doi: 10.1186/1752-0509-2-55 (PMC2446383; doi:10.1186/1752-0509-2-55)
Supplement: Additional file 1 — SBRT Archive. An archive of the current version of the Systems Biology Research Tool. [file 1752-0509-2-55-S1.zip › sbrt-1.4.0/doc/developers_guide/api/sbrt/kernel/math/algebra/IndexPair.html]

IndexPair


|  |  |  |
| --- | --- | --- |
| **Overview** \| **Package** \| **Class** \| **Use** \| **Tree** \| **Deprecated** \| **Index** \| **Help** | |  |
| **PREV CLASS**   **NEXT CLASS** | **FRAMES**    **NO FRAMES**     **All Classes** |  |
| SUMMARY: NESTED \| FIELD \| CONSTR \| METHOD | DETAIL: FIELD \| CONSTR \| METHOD |  |


---


## sbrt.kernel.math.algebra Class IndexPair<R,C>

```
java.lang.Object
  sbrt.kernel.math.algebra.IndexPair<R,C>
```

**Type Parameters:**: `R` - the row index type.: `C` - the column index type.

---

``` public final class IndexPair<R,C> extends java.lang.Object ```

This class is used to represent pairs of matrix indices.

**Author:**
:   This class was written and documented by
    Jeremiah Wright while in the Wagner lab.

---

**Constructor Summary**

`IndexPair(S rowIndex,
T columnIndex)`
  
          Constructs a new pair of matrix indices.


| **Method Summary** | |
| --- | --- |
| `boolean` | `equals(java.lang.Object obj)` |
| `C` | `getColumnIndex()`             Returns the column index. |
| `R` | `getRowIndex()`             Returns the row index. |
| `int` | `hashCode()`             Returns a content-based hash code. |

**Methods inherited from class java.lang.Object**

`clone, finalize, getClass, notify, notifyAll, toString, wait, wait, wait`

**Constructor Detail**

### IndexPair

```
public IndexPair(S rowIndex,
                 T columnIndex)
```

:   Constructs a new pair of matrix indices.

    **Type Parameters:**: `S` - a type extending the primary row type.: `T` - a type extending the primary column type. **Parameters:**: `rowIndex` - the row index.: `columnIndex` - the column index.


**Method Detail**

### getRowIndex

```
public R getRowIndex()
```

:   Returns the row index.

    :   **Returns:**: the row index.

---


### getColumnIndex

```
public C getColumnIndex()
```

:   Returns the column index.

    :   **Returns:**: the column index.

---


### hashCode

```
public int hashCode()
```

:   Returns a content-based hash code.

    :   **Overrides:**: `hashCode` in class `java.lang.Object`
    :   **Returns:**: a content-based hash code.

---


### equals

```
public boolean equals(java.lang.Object obj)
```

:   **Overrides:**: `equals` in class `java.lang.Object`


---


|  |  |  |
| --- | --- | --- |
| **Overview** \| **Package** \| **Class** \| **Use** \| **Tree** \| **Deprecated** \| **Index** \| **Help** | |  |
| **PREV CLASS**   **NEXT CLASS** | **FRAMES**    **NO FRAMES**     **All Classes** |  |
| SUMMARY: NESTED \| FIELD \| CONSTR \| METHOD | DETAIL: FIELD \| CONSTR \| METHOD |  |


---
